# High Selectivity Fuel from Efficient CO_2_ Conversion by Zn-Modified rGO and Amine-Functionalized CuO as a Photocatalyst

**DOI:** 10.3390/ma16124314

**Published:** 2023-06-11

**Authors:** Retno Damastuti, Diah Susanti, Adhimoorthy Prasannan, Wesley Wei-Wen Hsiao, Po-Da Hong

**Affiliations:** 1 Department of Materials Science and Engineering, National Taiwan University of Science and Technology, Taipei 106335, Taiwan; retnodamastuti4@gmail.com (R.D.); ak.prasannan@mail.ntust.edu.tw (A.P.); 2 Department of Materials and Metallurgical Engineering, Institut Teknologi Sepuluh Nopember (ITS), Surabaya 60111, Indonesia; santiche@mat-eng.its.ac.id; 3 Department of Chemical Engineering, National Taiwan University of Science and Technology, Taipei 106335, Taiwan

**Keywords:** CO_2_ conversion, rGO/CuO, photocatalyst, composite, fuel production

## Abstract

Reduced graphene oxide (rGO) has been used in copper (II) oxide (CuO)-based photocatalysts as an additive material. An application of this CuO-based photocatalyst is in the CO_2_ reduction process. The preparation of rGO by a Zn-modified Hummers’ method has resulted in a high quality of rGO in terms of excellent crystallinity and morphology. However, implementing Zn-modified rGO in CuO-based photocatalysts for the CO_2_ reduction process has yet to be studied. Therefore, this study explores the potential of combining Zn-modified rGO with CuO photocatalysts and performing these rGO/CuO composite photocatalysts to convert CO_2_ into valuable chemical products. The rGO was synthesized by using a Zn-modified Hummers’ method and covalently grafted with CuO by amine functionalization with three different compositions (1:10, 1:20, and 1:30) of rGO/CuO photocatalyst. XRD, FTIR, and SEM were used to investigate the crystallinity, chemical bonds, and morphology of the prepared rGO and rGO/CuO composites. The performance of rGO/CuO photocatalysts for the CO_2_ reduction process was quantitively measured by GC–MS. We found that the rGO showed successful reduction using a Zn reducing agent. The rGO sheet could be grafted with CuO particles and resulted in a good morphology of rGO/CuO, as shown from the XRD, FTIR, and SEM results. The rGO/CuO material showed photocatalytic performance due to the advantages of synergistic components and resulted in methanol, ethanolamine, and aldehyde as fuel with amounts of 37.12, 8730, and 17.1 mmol/g catalyst, respectively. Meanwhile, adding CO_2_ flow time increases the resulting quantity of the product. In conclusion, the rGO/CuO composite could have potential for large-scale CO_2_ conversion and storage applications.

## 1. Introduction

Global warming has resulted from greenhouse gas (e.g., CO_2_, methane) emissions brought on by the industrial revolution and an overreliance on fossil fuels for energy. Increased atmospheric CO_2_ concentration is the leading cause of global climate change [1]. Significantly, the CO_2_ concentration in the atmosphere contributes to various negative impacts on the environment and human health. To address this issue, researchers have explored various approaches to deal with CO_2_ emissions for sequestering excess CO_2_ from the atmosphere. Several approaches have been proposed for mitigating and resolving CO_2_ emission issues, including alternative energy sources, carbon capture storage (CCS), microbial conversion, electrochemical conversion, artificial photosynthesis, and photocatalysis. One promising approach has the dual-purpose advantage of decreasing the level of CO_2_ by converting it into valuable chemicals using photocatalyst materials [2]. 

Photocatalyst materials can absorb energy from light and then use it to trigger chemical reactions [3,4,5]. When exposed to sunlight, these materials can provide the energy needed by electron–hole transfer to drive CO_2_ conversion into various useful products, including methanol, ethanol, formaldehyde, and many other hydrocarbons [6]. Since Tooru Inoue et al. first reported the concept of semiconductor photocatalysts that can convert CO_2_ into valuable chemicals from solar energy, semiconductor materials have become promising materials for further related applications [7]. Over the past few decades, various photocatalyst materials have been developed, including metal, sulfides [8], metal oxides [9], graphene [10], polymeric materials [11], noble-metal-free [12,13], and metal–organic frameworks (MOFs) [14]. Among them, metal oxides offer high photoresponse, light absorption, and crystallinity, which are essential for photocatalytic activity.

Copper (II) oxide (CuO), a metal oxide semiconductor, offers low prices with many applications. The energy band gap of CuO (1.2 eV) can absorb and harvest visible light energy [15,16,17]. To this end, Biplab Singha et al. reported applying CuO/ZnO and CuO/TiO_2_ as photocatalyst materials. Moreover, adding CuO could enhance photocatalytic activity by increasing separation efficiency, which is related to electron–hole transfer from 3.3 to 7.2 and 6.1 to 8.1, respectively [18]. Minale et al. used CuO/ZnO in a chitosan matrix as a composite film to convert carbon dioxide, which yielded a methanol 2.16 mmol/g catalyst under ultraviolet light for six hours [19]. However, the potential efficiency of CuO as a photocatalyst material for CO_2_ conversion still has several challenges [20]. One of the main challenges is the low efficiency due to the fast electron–hole recombination of these materials [21]. Many efforts have been focused on enhancing photocatalytic performance efficiency by developing hybrid materials and optimizing reaction conditions [22]. Thus, carbon-based semiconductor nanocomposites have received great attention for obtaining improved efficiency in photocatalytic CO_2_ conversion. 

Remarkably, reduced graphene oxide (rGO) as a suitable material combined with metal oxide has gained increasing attention [23]. Huang et al. found that the large surface area and adjustable structure of rGO can effectively promote rapid electron mobility and decrease recombination, considerably improving photocatalytic performance [24]. Romeiro et al. discovered that the quality of rGO impacts photocatalytic activity under UV/Vis because adding rGO accelerates the charge transfer [25]. The quality of rGO depends on its synthesis method [26]. Hummers’ method is well known as a simple and inexpensive method to prepare graphene oxide (GO) [27]. It involves oxidizing graphite with harsh acid at a low temperature, followed by ultrasonication to exfoliate the layer of GO sheet, and then reduction with hydrazine hydrate to obtain rGO [28]. However, hydrazine hydrate has limitations due to its poisonous nature and high toxicity, leading to undesirable defects of GO and flammability [29]. Yang et al. prepared high-quality rGO using a modified Hummers’ method by adding Zn as an alternative reducing agent, which is non-toxic, low-cost, and relatively easy to handle, to obtain a higher degree of reduction and better stability in the solution [30]. Rashi et al. [31] showed that CO_2_ can be converted to 0.17 mmol/g methanol using a pure CuO photocatalyst. Adding rGO to the CuO photocatalyst formed an rGO/CuO photocatalyst composite with a constant ratio of 1:20, increasing the methanol yield to 1.22 mmol/g, and rGO/CuO managed to show good photocatalytic performance. Table 1 shows a summary of the stability and product yield of rGO/CuO-based composites. However, the conventional method for preparing rGO resulted from Hummers’ reduction process with hydrazine hydrate as the reducing agent. In addition, variations in the composition of rGO/CuO composites on photocatalyst performance still need to be studied. Therefore, studies on synthesizing rGO by Hummers’ reduction process with the reducing agent zinc (Zn) and implementing this synthesized rGO into rGO/CuO composite photocatalysts with various compositions need to be conducted.

In brief, this study aimed to develop hybrid materials by synthesizing rGO (via the modified Hummers’ method) and covalently grafting rGO with CuO to create composites as a photocatalyst to convert CO_2_ into different fuels efficiently. Our results indicated that these hybrid materials significantly enhanced the photocatalytic products’ efficiency. Furthermore, this study investigated the effect of CuO composition and CO_2_ gas flow time on the rGO/CuO composite as the photocatalyst material for converting CO_2_ to valuable chemicals and fuels under visible irradiation to reduce CO_2_ emission.

## 2. Materials and Methods

### 2.1. Materials

Graphite powder (Purity 99%), NaNO_3_, KMnO_4_, H_2_O_2_, NH_4_OH, HCl, BaCl_2_, NaOH, Zn powder, Cu(CO_2_CH_3_)_2_•H_2_O, Cu(NO_3_)_2_•H_2_O, 3-aminopropyltrimethoxysilane (APTMS) DMF, and H_2_SO_4_ were supplied by Merck Sigma-Aldrich with analytical grade.

### 2.2. Synthesis Process of rGO

The rGO was produced using Hummers’ method and modified by adding Zn powder as a reducing agent, which offers good stability in the solution to exfoliate GO^30^.

Firstly, we prepared GO by stirring 2 g of graphite powder in 80 mL of 98% H_2_SO_4_ solution at an ice bath temperature (0–5 °C) for 4 h. While the stirring process ran for 2 h, we gradually added 4 g NaNO_3_ and 8 g KMnO_4_ into the solution. The addition of KMnO_4_ made the solution greenish-black. Then, we removed the solution from the ice bath and stirred it at 35 °C for 20 h. Next, 20 mL of H_2_O_2_ was added for 30 min to remove the remaining KMnO_4_ content in the solution (turning it bright yellow). Later, the solution was centrifuged for 1 h at 2500 rpm to separate the solution, and then yellow solids were obtained, while colorless liquids were not used. We washed the yellow solid with 5% HCl (0.01 M) to remove the remaining metal ions. We used DI water continuously to neutralize the pH of the solid. Titration was conducted with 1M BaCl_2_ to test whether the sulfate ions had disappeared until the pH was neutral. During the BaCl_2_ titration, the drying process was conducted at 110 °C for 12 h, and, finally, GO powder was obtained.

Subsequently, 40 mg of the produced GO powder was dissolved into 40 mL of DI water and stirred for 1 h for a homogeneous GO solution. The ultrasonication of the solution for 1.5 h was conducted to obtain GO. In the next step, we added 10 mL of 37% HCl in non-stirring conditions. The reduction process was conducted using a Zn-reducing agent of 1.6 g. After all the bubbles were gone, we added 10 mL of 37% HCl and waited 30 min to homogenize the solution and remove the remaining Zn. Then, the black precipitate was washed with DI water several times until the pH became neutral. Finally, the precipitate was put into the autoclave for a hydrothermal process at 160 °C for 12 h to produce rGO.

### 2.3. Synthesis Process of CuO

CuO was mixed with 0.0125 mol Cu(CO_2_CH_3_)_2_•H_2_O and 0.3 mol NaOH solution at room temperature for two hours with continuous stirring (turning from green to blue when mixing with NaOH). After centrifugation, the collected sample was washed three times using DI water and ethanol as a closing solution.

### 2.4. Synthesis of rGO/CuO

**Figure 1:**
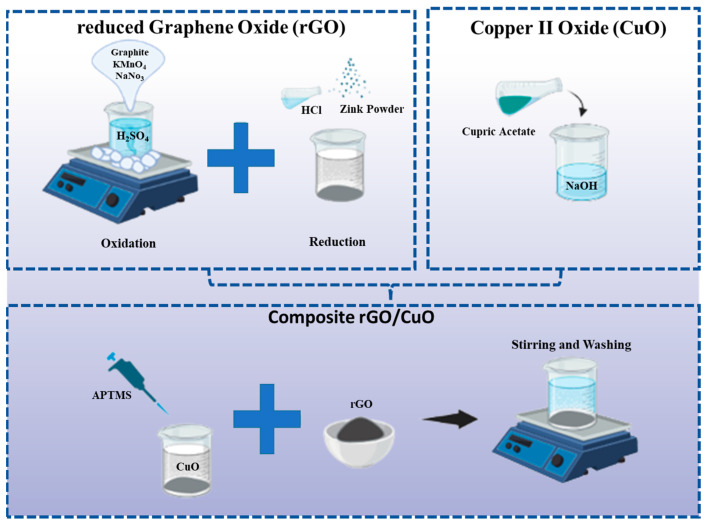
Schematic illustration of rGO/CuO composite preparation.

CuO was dissolved into toluene and purged by nitrogen gas to remove contaminants. Then, the solution was added with 1.5 mL of APTMS and flushed with nitrogen overnight with continuous stirring. After the mixture was cooled to room temperature, we washed the mixture with toluene, ethanol, and DI water. The APTMS and CuO mixtures have amino functional groups that can bind to rGO. To this end, CuO-APTMS was added with rGO at 80 °C and stirred for 15 h at pH 10. Finally, after washing with water and ethanol, the mixture was dried at 110 °C for 6 h. Rashi et al. reported using a ratio of rGO/CuO composite of 1:20 for the photocatalytic conversion of CO_2_ into methanol under visible light irradiation [31]. Thus, to observe the photocatalytic efficiency, this study investigated different rGO/CuO nanocomposite ratios of 1:10, 1:20, and 1:30. Figure 1 illustrates the entire procedure.

### 2.5. Characterization

X-ray diffraction (XRD) was used to determine the crystal structure using a Panalytcal machine (Malvern Panalytical Ltd., Cambridge, UK). A Scimitar 1000 Fourier Transform Infrared (FTIR) machine (Agilent Technologies Inc., Palo Alto, CA, USA) was employed to determine composites’ functional groups and chemical bonds. A scanning electron microscope (SEM) was also used to analyze particle morphology and size with the SEM Inspect S50 instrument (FEI Company, Hillsboro, OR, USA). Gas chromatography–mass spectroscopy (GC–MS) was used to qualitatively measure CO_2_ conversion products using the GCMS Agilent 6980N Network GC System with an autosampler and detector using an Agilent 5973 inert MSD (Agilent Technologies, Inc., Santa Clara, CA, USA).

### 2.6. Experimental Setup for CO_2_ Conversion

**Figure 2:**
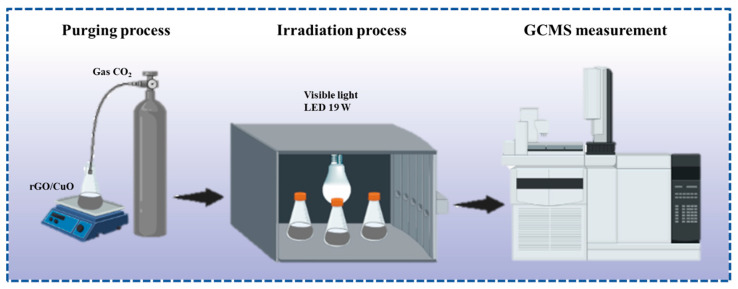
Diagram of the experimental setup for CO_2_ conversion.

The CO_2_ reduction experiments were carried out in blank, 1:10, 1:20, and 1:30 photocatalyst ration solutions of rGO/CuO. The blank sample is a 45/5 mL DMF (Dimethylformamide)/H_2_O solution without photocatalyst material. DMF was chosen as a suitable solvent to induce the polarity of the catalyst. The photocatalyst solutions were prepared by adding and stirring 100 mg rGO/CuO composites in 45/5 mL DMF/H_2_O solutions for an hour. After that, nitrogen gas was purged for 15 min to remove other gases before the CO_2_ reduction process. Under continuous stirring, CO_2_ gas was purged into each composition variable of the solution to make saturated solutions. The CO_2_ gas flow were varied 5, 10, and 15 min. So, the total samples are 12 with different composition catalysts and CO_2_ gas flows. All saturated solutions were shielded and irradiated with visible light using a 19 W LED lamp inside a closed box for 24 h. The distance between the saturated solution and the light source was 5 cm during radiation. After the radiation process, the sample was collected and tested by GC–MS. Each sample was tested three times. The detailed process is shown in Figure 2.

## 3. Results and Discussions

### 3.1. Formation of rGO/CuO Composite

**Figure 3:**
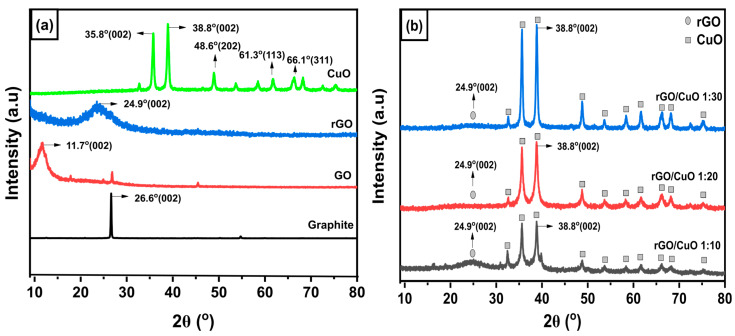
XRD Pattern for (**a**) graphite, GO, rGO, and CuO, (**b**) rGO/CuO with composition 1:10, 1:20, and 1:30.

The formation of the rGO/CuO nanocomposite was identified by an analysis of its crystalline nature through the XRD pattern. As shown in Figure 3, rGO was successfully formed through the modified Hummers’ method, and rGO/CuO nanocomposite was obtained through the amine-functionalized CuO and rGO surface functional groups through their crystalline nature changes. A high peak of graphite indicated high crystallinity from graphite flakes. Figure 3a gives evidence of the transformation of graphite into GO. This transformation is depicted by peak shifting from 2Ɵ of 26.6° at the plane of (002), indicated as the peak of graphite according to JCPDS no 03-065-6212, to the 2Ɵ of 11.7° at the plane of (002), indicated as a peak of GO. The oxidation process yielded GO and retained a small amount of graphite. After that, the reduction process by adding Zn powder as a reduction agent resulted in rGO, which was confirmed by a new amorphous pattern 2Ɵ of 24.9° at the plane of (002). The XRD shifting peaks of graphite, GO, and rGO were also mentioned by Phukan et al. [37]. According to JCPDS no 801917, CuO has a monoclinic crystal structure. The CuO sample exhibited two sharp peaks at 2Ɵ of 35.8° at the plane (002) and 2Ɵ of 38.8° at the plane (002). Other CuO’s peaks with lower intensities were indicated at 2Ɵ of 48.6° at the plane of (202), 2Ɵ of 61.3° at the plane of (113), and 2Ɵ of 66.1° at the plane of (311) [38].

Figure 3b shows the XRD pattern of the rGO/CuO composite with three ratios of 1:10, 1:20, and 1:30, respectively. In the rGO/CuO composite, there is an amorphous rGO peak at 24.9°, which indicates that the composition of rGO was identified at a ratio of 1:10. Meanwhile, in the rGO/CuO composites with ratios of 1:20 and 1:30, the peak intensity of rGO decreased as the amount of CuO in the composite increased. 

The interlayer distance of some phases, including graphite, GO, rGO, and CuO, were calculated from Bragg’s Law using the following Equation (1).

(1) $$d_{hkl}=\frac{\lambda }{2\sin\theta }$$

where *d_hkl_* was the interlayer distance (Å) of each observed phase at their highest XRD’s peak and their specific plane, *λ* was CuKα’s wavelength (1.54 Å), and *θ* was the diffraction angle (°).

**Figure 4:**
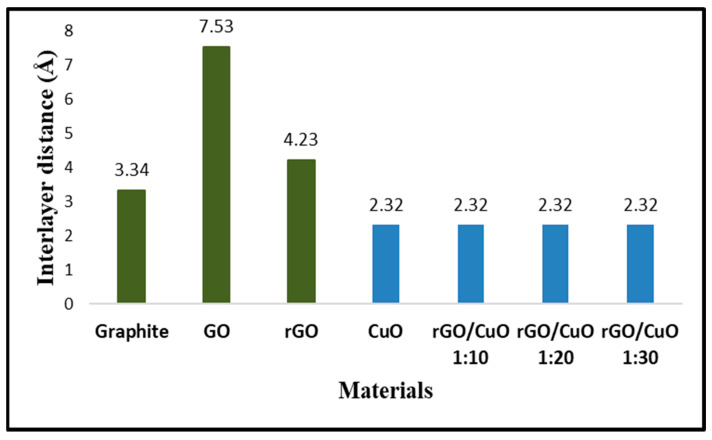
Calculation of the distance between layers of graphite, GO, rGO, CuO, and rGO/CuO composites.

The interlayer distances of the graphite, GO, and rGO are exhibited by the green bars, while the interlayer distances of CuO before and after rGO addition are shown by the blue bars of Figure 4. The purpose of calculating *d_hkl_* is to understand changes in the interlayer distance during the formation reaction process of graphite, GO, and rGO. Initially, the precursor was graphite, which has an interlayer distance of 3.34 Å at 2Ɵ of 26.6° with a plane of (002). After undergoing an oxidation process, graphite turns into GO. The calculation results show that GO has an interlayer distance of 7.53 Å at 2Ɵ of 11.7° with the plane of (002). An increase in this value indicates the success of the oxidation process, which means the formation of functional groups that insert into GO’s interlayers, resulting in a wider distance between its layers. In addition, GO was reduced using Zn powder to become rGO, which causes the interlayer distance to be 4.23 Å at 2Ɵ of 24.9° with the plane of (002). This phenomenon shows that the reduction process decreases the pre-existing functional groups. Therefore, the distance of rGO’s interlayer becomes narrower.

Furthermore, the interlayer distance was calculated on CuO to determine the effect of rGO addition in CuO interlayers. The XRD peak of CuO at 2Ɵ of 38.8° with the plane (002) exhibited an interlayer distance of 2.32 Å. After CuO was treated using APTMS then grafted with rGO to become an rGO/CuO composite, the XRD peak of CuO at 2Ɵ of 38.8° with the plane (002) showed the value of the interlayer distance as 2.32 Å. The interlayer distance in each rGO/CuO composite with compositions of 1:10, 1:20, and 1:30 shows the same value. It was indicated that there is no doping of the molecule rGO into the internal layer of the CuO molecules, which did not cause an enlargement of the CuO interlayer distance. However, rGO only interacts chemically to bind with CuO to form rGO/CuO composites with intermolecular interactions. The FTIR result will explain the interaction in the rGO/CuO composite in more detail.

### 3.2. The Formation of Interaction in the rGO/CuO Composite

**Figure 5:**
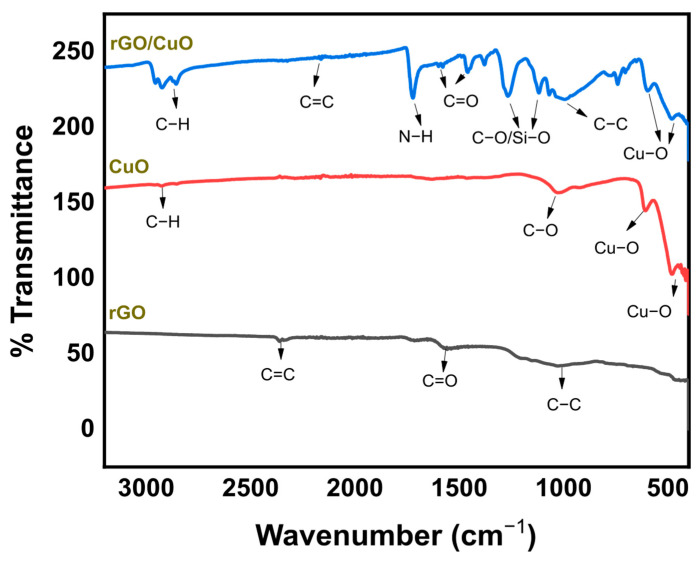
FTIR spectra of rGO, CuO, and rGO/CuO composites.

FTIR testing was carried out on samples of rGO, CuO, and rGO/CuO composites to determine the types of bonds present in each of the rGO and CuO phases, as well as the molecular interactions in the rGO/CuO composites, as shown in Figure 5. The rGO shows vibrations at wavenumbers 2323 cm^−1^, 1636 cm^−1^, and 1036 cm^−1^, respectively, indicating the presence of C=C, C=O, and C–C bonds. Meanwhile, in the CuO, there is a strong vibration at the wavenumber range of 400–600 cm^−1^, indicating a monolithic crystal structure of Cu–O bonds. At wavenumbers 1030 cm^−1^ and 2924 cm^−1^, there are C–O and C–H bonds, indicating that CuO was synthesized from copper acetate and still left unreacted precursors. The residues in the form of C=O bonds in rGO, C–O, and C–H in CuO were used for the rGO/CuO grafting process with the help of APTMS. In the rGO/CuO composite, vibrations at wavenumbers 2195 cm^−1^, 1636 cm^−1^, and 1036 cm^−1^ indicate the C=C, C=O, and C–C bonds of rGO [39]. CuO in the composite is indicated at the peak with the wavenumber range of 600–500 cm^−1^. APTMS grafting CuO with rGO is indicated by the presence of Si–O, C–O, and N–H groups at wavenumbers 1100 cm^−1^, 1000 cm^−1^, and 1598 cm^−1^. The interaction bonds of Si–O and C–O at wavenumber 1100–1000 cm^−1^ resulted from chemical bonds between APTMS-CuO and APTMS-rGO in the rGO/CuO composite.

### 3.3. The Morphology of the Formation of the rGO/CuO Composite

**Figure 6:**
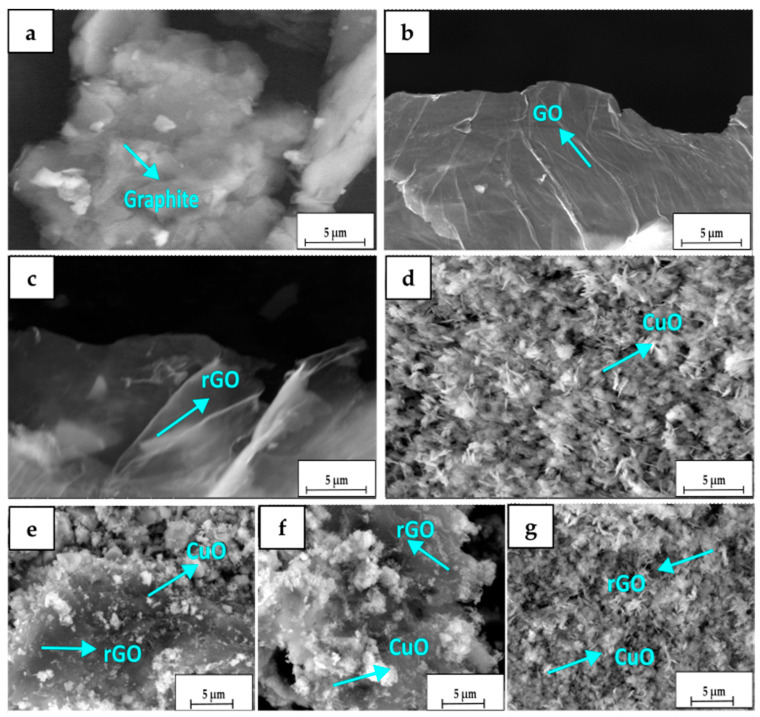
SEM images of (**a**) graphite, (**b**) GO, (**c**) rGO, and (**d**) CuO and rGO/CuO composite with a ratio of weight of (**e**) 1:10, (**f**) 1:20, and (**g**) 1:30.

The SEM analysis determined the surface morphology and structural differences of the graphite, GO, rGO, CuO, and rGO/CuO composite. Figure 6a shows the SEM results on a graphite sample with small and irregular particles with flake-like morphology. After the oxidation of graphite, the morphology became layered sheets, as shown in Figure 6b. In the morphology shown in Figure 6c, the rGO was formed of thinner sheets than the GO due to the chemical exfoliation process. Figure 6d exhibits the morphology of CuO with the agglomerated leaf-like structure [40].

To understand the influence of the surface morphology of rGO/CuO, various compositions, such as 1:10, 1:20, and 1:30, were performed. Figure 6e–g shows that CuO particles are tiny particles bonded to rGO sheets due to their intermolecular interactions, confirmed in the FTIR and XRD results. The composition ratio of 1:20 was more prominent and bound to the rGO sheets. The higher composition of 1:30 shows that the CuO particles are homogeneously distributed throughout the rGO surface without rGO visible due to its low concentration, and that it was masked by a large amount of CuO particles. Overall, the composite morphology indicated the existence of rGO and CuO. 

### 3.4. The Quantification of Fuel as Resulting from CO_2_ Conversion

The synergic effects between amine-functionalized CuO nanoparticles and rGO efficiently contribute to the photoconversion of CO_2_. To understand rGO/CuO composite photocatalytic performance, we dispersed the composite into DMF/H_2_O, in which nitrogen was previously purged to remove other gases, and then CO_2_ was purged at varying times for each sample. DMF was used as the solvent for its good polar dispersion with matching for rGO preparation [41]. The sample was irradiated with a visible light 19 W LED lamp (Philips) for 24 h at a 5 cm distance from the light source. After irradiation, a 1 µL sample in the liquid phase was injected into the GC–MS to evaluate the product from photoconversion from the composite. The results of the GC–MS analysis show that carbon dioxide (CO_2_) was converted into several kinds of fuels. Chemical reactions upon irradiation with catalysts may lead to a particular time and condition. The results of CO_2_ conversion via a photocatalytic process produced various fuels, such as methanol, ethanolamine, and aldehyde, in the presence of the composite catalyst rGO/CuO. The quantitative analysis of products produced using GC–MS is described in the Appendix A.

**Figure 7:**
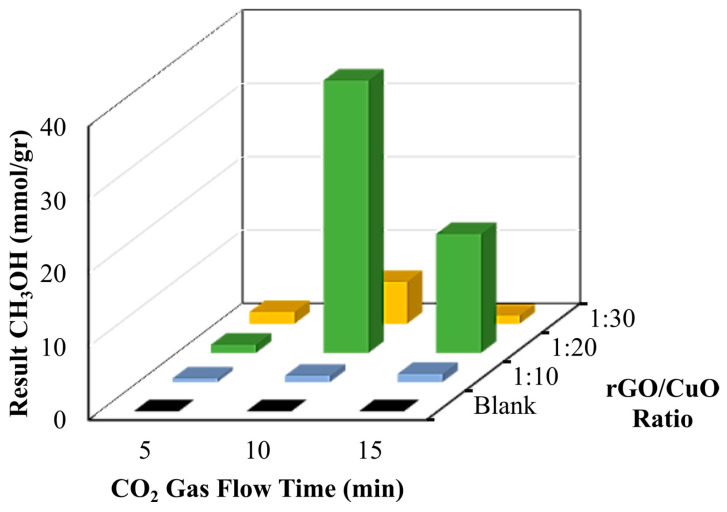
Calculation results of CO_2_ that has been converted to methanol.

Moreover, Figure 7 and Table 2 show the amount of methanol as a product of the conversion of CO_2_ gas with various rGO/CuO composite catalysts under different times of CO_2_ flow. The rGO/CuO mixed catalyst with a weight composition ratio of 1:10 produced 0.49 mmol/g methanol for 5 min, 0.86 mmol/g methanol for 10 min, and 1.11 mmol/g methanol for 15 min, respectively. Furthermore, the rGO/CuO composite catalyst with a weight composition ratio of 1:20 produced different amounts of methanol. When the CO_2_ was purged for 5 min, 1.11 mmol/g methanol was produced. When CO_2_ was purged for 10 min, 37.12 mmol/g methanol was produced. However, the methanol conversion efficiency decreased when the time was increased to 15 min. Note that the rGO/CuO composite catalyst with a weight composition ratio of 1:30 showed the trend of converting CO_2_ into methanol with lower efficiency than 1:20 compositions. 

Overall, the results of the GC–MS analysis indicated that a photocatalytic reaction with an rGO/CuO catalyst, which was activated using light, had occurred for all Erlenmeyer tubes containing DMF/H_2_O solution samples and produced a certain amount of methanol. The highest amount of methanol was found in the rGO/CuO composite with a composition of 1:20 with a CO_2_ gas flow time of 10 min and then followed by a CO_2_ gas flow time of 15 min. The significant difference in results occurred due to the methanol being oxidized so that the quantity of the produced methanol decreased. Likewise, the DMF/H_2_O solution with an rGO/CuO catalyst with a composition of 1:30 has a maximum yield of CO_2_ gas flow time at 10 min, which was 5.69 mmol/g methanol, and decreased at 15 min to produce 1.11 mmol/g methanol. In brief, the composites with rGO/CuO of 1:10 generally showed that the more CO_2_ flow gas time increases, the more CO_2_ has been converted to produce ethanolamine.

**Figure 8:**
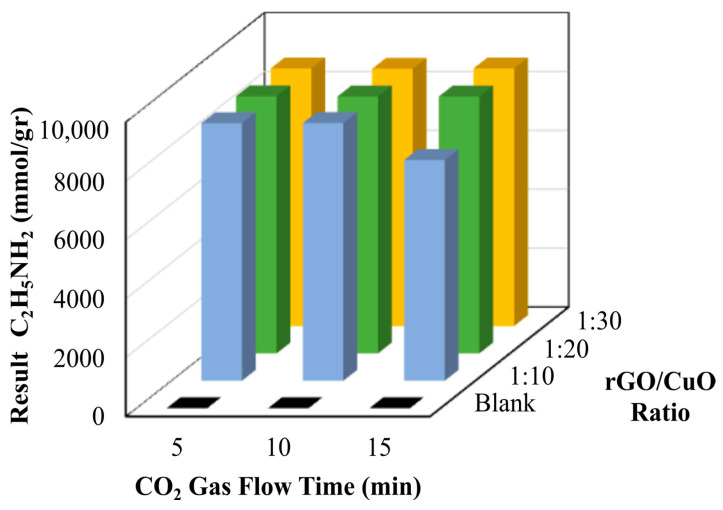
Calculation results of CO_2_ that has been converted to ethanolamine.

The photocatalytic conversion of ethanolamine from CO_2_ to understand the efficiency of CO_2_ utilization is shown in Figure 8 and Table 3. The DMF/H_2_O solution with the rGO/CuO catalyst variations in the weight composition ratio of 1:10 at CO_2_ gas flow time for 5 min produces 8754 mmol/g ethanolamine. When CO_2_ was purged for 10 min, 8757 mmol/g of methenamine was made. When CO_2_ was purged for 15 min, 7501 mmol/g ethanolamine was produced. These results differed from the rGO/CuO composite catalyst with variations in the composition weight ratio of 1:20 at CO_2_ gas flow time for 5 min to produce 8740 mmol/g ethanolamine, merely exhibiting independent conversion with CO_2_ gas flow for 10 or 15 min, respectively. An equivalent efficiency was obtained with the rGO/CuO composite catalyst at a ratio of 1:30, producing approximately 8751 mmol/g of ethanolamine in CO_2_ flow. The yield of ethanolamine increased with increasing CO_2_ gas flow time and decreased again when the CO_2_ was purged for 15 min. The high quantity of ethanolamine was obtained due to the reaction between ethanol and nitrogen-based functional groups. The nitrogen-based functional groups were attributed to the presence of the -NH_2_ (amine) functional group in APTMS and the -N-C (nitrile) functional group in the DMF. These functional groups react with the ethanol and result in ethanolamine.

**Figure 9:**
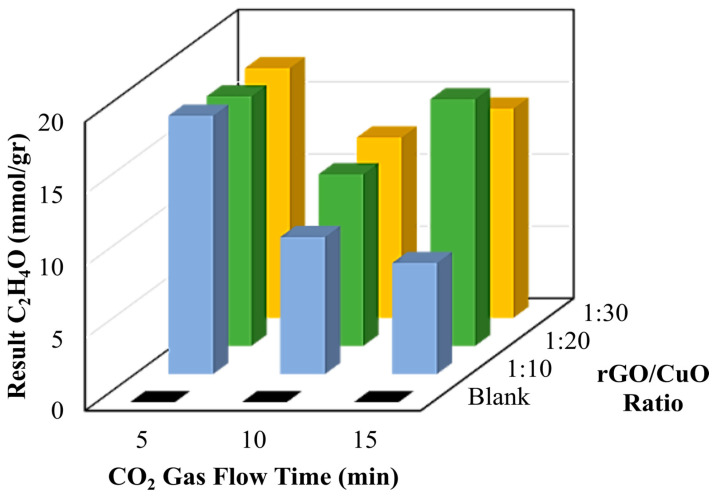
Calculation results of CO_2_ that has been converted to formaldehyde.

Moreover, the obtained catalyst was successfully applied to convert formaldehyde from methanol oxidation, and the amount of formaldehyde production is shown in Figure 9 and Table 4. To measure the effects of the catalyst and CO_2_ gas flow time on the formaldehyde conversion, an rGO/CuO composite with 1:10 was subjected to various CO_2_ gas flow times of 5, 10, and 15 min and produced 17.9, 9.5, and 7.7 mmol/g aldehyde, respectively. In the case of 1:20, formaldehyde conversion was monitored as 17.3, 11.9, and 17.1 mmol/g aldehyde for 5, 10, and 15 min flow time. A similar trend has been observed for the 1:30, with about 17.3, 12.5, and 14.5 mmol/g formaldehyde upon the 5, 10, and 15 min flows, respectively. The photocatalytic conversion of CO_2_ to valuable fuels, such as methanol, ethanolamine, and formaldehyde, is effectively converted upon light irradiation. Conversation efficiency may occur from the synergistic effects between rGO and CuO.

### 3.5. Photocatalytic Conversion Mechanism

The photocatalytic conversion starts when the photocatalyst is irradiated with light of the appropriate wavelength. An electron jumps from the conduction band, leaving a positive hole in the valence band. Electrons in the conduction band can reduce other substrates, while gaps in the valence band can oxidize some compounds [42]. In the conduction band, the electrons react with oxygen from H_2_O reduction to form superoxide anion species (O_2_^−^). Thus, superoxide anions can react with compounds (CO_2_) resulting from the breakdown of organic molecules to develop products. Meanwhile, a hole will be produced in the valence band. Holes will split water to form a hydroxyl radical (OH^.^). The hydroxyl radicals then react with organic molecules and break down these organic compounds into other intermediate compounds undergoing further reactions [43]. This cycle continues to repeat until the reaction is completed. 

**Figure 10:**
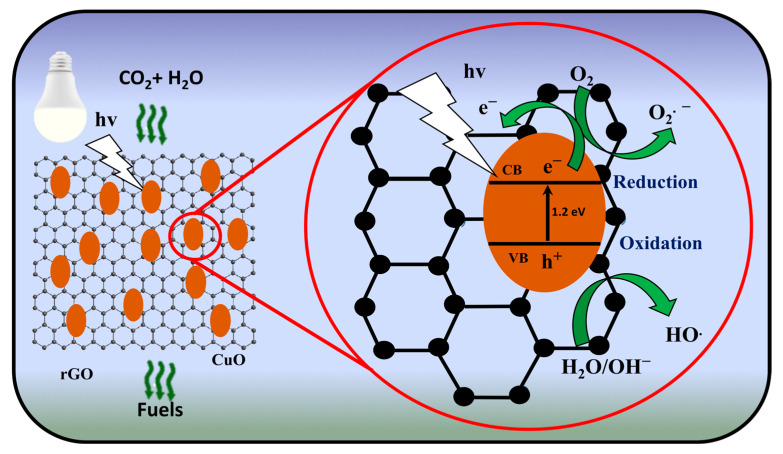
Schematic photocatalytic mechanism of CO_2_ conversion.

However, the generation of electron–hole pairs is accompanied by the risk of recombination, where the free electron and the hole combine and return to the valence band, reducing the photocatalytic activity [44]. To prevent the recombination phenomenon, it is necessary to suppress the recombination rate of the electron–hole pairs, which can be achieved by introducing a cocatalyst or by designing the photocatalyst with appropriate bandgap and band positions [45]. The CuO has band gap 1.2 eV [46]. Meanwhile, rGO acts as a cocatalyst to suppress the recombination of electron–hole pairs by providing a pathway to transfer electrons from the conduction band of CuO with the rGO surface, effectively trapping the electrons and preventing their recombination with holes in the valence band [47]. The rGO surface also provides a large surface area for CO_2_ adsorption and the generation of active sites for converting CO_2_ [48]. In another approach from the direct pathway, CO_2_ molecules have been reduced directly to the desired product by the free electrons. On the other hand, in the indirect path, CO_2_ molecules have been firstly converted to intermediate species, which were then reduced to the desired products by the free electrons. To summarize, the possible conversions are listed in Table 5, and the schematic illustration is shown in Figure 10.

CuO can absorb visible light and uses photon energy to excite electrons and reduce CO_2_ (DMF/H_2_O as solvents into valuable fuels). However, the recombination of electrons in the initial stages is a challenge because of the narrow band gap of CuO. In addition to obtaining high electrical conductivity, rGO sheets provide electrons in CuO to transfer to rGO sheets when excited by photons. Hence, the electrons can be used in CO_2_ reduction. To this end, the amount of rGO strongly influences the performance of the CuO photocatalyst as a support for electron transfer.

Moreover, the morphology of CuO, especially its porosity, influences photocatalytic performance. Photocatalyst performance can be reduced by a lack of CuO, such as a rGO/CuO ratio of 1:10, because the rGO sheet can completely cover the CuO porous surface. The porous CuO should absorb the CO_2_ molecules and solvents to facilitate the reduction process. The optimum amount of CuO, such as an rGO/CuO ratio of 1:20, leads rGO to be an optimal facilitator for accelerating electron transfer and providing sufficient porosity in the catalyst for the CO_2_ gas reduction. Meanwhile, when the amount of CuO is too much, as in the ratio of rGO/CuO 1:30, it leads excited electrons to recombine easily, and so too were electrons not enough for the CO_2_ gas reduction process.

## 4. Conclusions

Photocatalytic materials from an rGO/CuO composite have been demonstrated as effective materials as photocatalysts for converting CO_2_ into valuable fuels and chemical products due to their acceleration of chemical reactions when exposed to visible light. Here, the rGO was obtained from Hummers’ modified method by adding Zn as a reducing agent, which was chemically linked with amine-functionalized CuO particles. The excellent photocatalytic performance of the rGO/CuO material is activated by the synergistic effects between chemically linked CuO and rGO. The ratio between rGO and CuO highly influences the production of valuable fuels, such as methanol, ethanolamine, and formaldehyde. The GC–MS test results showed that the rGO/CuO 1:20 composite for 10 min of CO_2_ gas flow produced 37.12 mmol/g, 8730 mmol/g, and 11.9 mmol/g of methanol, ethanolamine, and formaldehyde, respectively. The rGO/CuO composite could have potential for large-scale CO_2_ conversion into valuable fuels.

## Figures and Tables

**Table 1:** Summary of the stability and product yield of rGO/CuO-based composites.

Composite	Stability	Application	Result	Ref.
rGO/CuO	6 cycle	CO_2_ conversion	Methanol 1.2 mmol/g	[31]
CeO_2_/rGO/CuO	5 cycle	CO_2_ conversion	Methanol 135.6 μmol g^−1^	[32]
Cu_2_O/RGO	6 cycle	CO_2_ conversion	CO 0.344%	[33]
PVA/rGO/(Cu_2_O/CuO)	4 cycle	CO_2_ conversion	Methanol 19.5 µmol cm^−2^ h^−1^	[34]
Cu-Zn N-dop rGO	6 cycle	CO_2_ conversion	Ethanol 195 µmol L^−1^h^−1^	[35]
CuO/Cu_2_O/CILE	5 cycle	CO_2_ conversion	Formaldehyde 110 mmol L^−1^	[36]

**Table 2:** Calculation results of CO_2_ that has been converted to methanol.

Ratio rGO/CuO	Time Flow CO_2_ (min)	Normalization (%)	Methanol Result (mmol/gr Catalyst)
Blank	5	0 ± 0	0 ± 0
	10	0 ± 0	0 ± 0
	15	0 ± 0	0 ± 0
1:10	5	0.004 ± 0.0005	0.49 ± 0.062
	10	0.007 ± 0.0008	0.86 ± 0.107
	15	0.009 ± 0.0012	1.11 ± 0.157
1:20	5	0.009 ± 0.0016	1.11 ± 0.201
	10	0.3 ± 0.036	37.12 ± 4.572
	15	0.131 ± 0.0264	16.21 ± 3.267
1:30	5	0.013 ± 0.0021	1.6 ± 0.324
	10	0.046 ± 0.0047	5.69 ± 0.765
	15	0.009 ± 0.0009	1.11 ± 0.175

**Table 3:** Calculation results of CO_2_ that has been converted to ethanolamine.

Ratio rGO/CuO	Time Flow CO_2_ (min)	Normalization (%)	Ethanolamine Result (mmol/gr Catalyst)
Blank	5	0 ± 0	0 ± 0
	10	0 ± 0	0 ± 0
	15	0 ± 0	0 ± 0
1:10	5	99.859 ± 12	8754 ± 1563.245
	10	99.894 ± 14	8757 ± 1458.92
	15	85.568 ± 9	7501 ± 1109.834
1:20	5	99.704 ± 10	8740 ± 1323.312
	10	99.591 ± 9	8730 ± 1450.056
	15	99.589 ± 15	8730 ± 1220.033
1:30	5	99.827 ± 12	8751 ± 987.536
	10	99.683 ± 12	8738 ± 1325.55
	15	99.823 ± 11	8751 ± 989.976

**Table 4:** Calculation results of CO_2_ that has been converted to formaldehyde.

Ratio rGO/CuO	Time Flow CO_2_ (min)	Normalization (%)	Formaldehyde Result (mmol/gr Catalyst)
Blank	5	0 ± 0	0 ± 0
	10	0 ± 0	0 ± 0
	15	0 ± 0	0 ± 0
1:10	5	0.2 ± 0.01	17.9 ± 3.12
	10	0.107 ± 0.0155	9.5 ± 1.5
	15	0.087 ± 0.0088	7.7 ± 1.205
1:20	5	0.194 ± 0.0176	17.3 ± 2.54
	10	0.133 ± 0.0155	11.9 ± 2.105
	15	0.192 ± 0.0177	17.1 ± 2.502
1:30	5	0.194 ± 0.0165	17.3 ± 2.534
	10	0.14 ± 0.017	12.5 ± 2.143
	15	0.163 ± 0.0183	14.5 ± 2.103

**Table 5:** Reaction of CO_2_ conversion during rGO/CuO photocatalyst activity.

Thermodynamic Electrochemical Reactions	E° (V vs. SHE)	Product
CO_2_(g) + 2H_2_O(l) + 2e− = HCOO− (aq) + OH−	−1.078	Formic Acid
CO_2_(g) + 2H_2_O(l) + 2e− = CO(g) + 2OH−	−0.934	Carbon Monoxide
CO_2_(g) + 3H_2_O(l) + 4e− = CH_2_O(l) + 4OH−	−0.898	Formaldehyde
CO_2_(g) + 5H_2_O(l) + 6e− = CH_3_OH(l) + 6OH−	−0.812	Methanol
CO_2_(g) + 6H_2_O(l) + 8e− = CH_4_(g) + 8OH−	−0.659	Methane
2CO_2_(g) + 9H_2_O(l) + 12e− = CH_3_CH_2_OH(l) + 12OH−	−0.744	Ethanol
H_2_O(l) + 2h+ = ½ O_2_ + 2H+	0.82	Oxygen

## Data Availability

The data presented in this study are available upon request from the corresponding author.

## Appendix A

An example of the methanol calculation from a photocatalytic reaction using the rGO/CuO composite with a composition ratio of 1:20 and a CO_2_ flow time of 10 min.

Mass rGO/CuO catalyst	= 0.1 g
vol. Injection	= 1 µL = 10^−3^ mL
Normalization CH_3_OH	= 0.3 vol % (resulted from GC–MS)
ρ CH_3_OH	= 0.792 g/cm^3^
Mr CH_3_OH	= 32
vol. CH_3_OH	= Normalization CH_3_OH × vol. Injection
	= 3% × 1 µL = 0.003 µL = 0.003 × 10^−3^ cm^3^
Mass CH_3_OH	= ρ CH_3_OH × vol. CH_3_OH
	= 0.792 g/cm^3^ × 0.003 × 10^−3^ cm^3^
	= 2.376 × 10^−6^ g
n CH_3_OH in solution	= Mass CH_3_OH/Mr CH_3_OH
	= 2.376 × 10^−6^ g/32
	= 7.425 × 10^−8^ mol = 0.07425 µmol
vol. Solution	= 50 mL
n CH_3_OH in injector	= (n CH_3_OH in solution × vol. solution)/vol. Injection
	= 0.07425 µmol × 50 mL/10^−3^ mL
	= 3712.5 µmol
n CH_3_OH/g catalyst	= n CH_3_OH in injector/Mass rGO/CuO catalyst
	= 3712.5 µmol/0.1 g
	= 37125 µmol/g = 37.125 mmol/g

Therefore, the result of methanol is 37.125 mmol/g catalyst.
